# ‘*Candidatus* Liberibacter brunswickensis’ colonization has no effect to the early development of *Solanum melongena*

**DOI:** 10.1038/s41598-024-66352-y

**Published:** 2024-08-02

**Authors:** Jacqueline Morris, Rachel Mann, Angage Sanka Perera, Rebekah Frampton, Mallik Malipatil, Sorn Norng, Alan Yen, Grant Smith, Brendan Rodoni

**Affiliations:** 1 Plant Biosecurity Cooperative Research Centre, LPO Box 5012, Bruce, Australian Capital Territory 2617 Australia; 2 Applied Systems Biology, AgriBio, La Trobe University, 5 Ring Road, Bundoora, VIC 3083 Australia; 3 Agriculture Victoria Research, AgriBio, Agriculture Victoria, 5 Ring Road, Bundoora, VIC 3083 Australia; 4 Australian Animal Health Laboratories, Australian Centre for Disease Preparedness, Commonwealth Scientific and Industrial Research Organisation (CSIRO), Geelong, VIC 3219 Australia; 5 The New Zealand Institute for Plant & Food Research Limited, Gerald St, Lincoln, 7608 New Zealand

**Keywords:** ‘*Candidatus* Liberibacter brunkswickensis’, CLbr, *Acizzia solanicola*, Psyllid, Eggplant, Colonisation, Transmission, Genetic techniques, Microbiology techniques

## Abstract

This study is the first to investigate the presence and movement of the novel Liberibacter species ‘*Candidatus* Liberibacter brunswickensis’ (CLbr) in eggplant, *Solanum melongena*. The psyllid, *Acizzia solanicola* can transmit CLbr to eggplant and CLbr can be acquired by CLbr-negative *A. solanicola* individuals from CLbr-positive eggplants. *In planta*, CLbr can replicate, move and persist. Investigation into the early development of eggplants showed that CLbr titres had increased at the inoculation site at 14 days post inoculation access period (DPIAP). CLbr had become systemic in the majority of plants tested by 28 DPIAP. The highest bacterial titres were recorded at 35 DPIAP in all samples of the inoculated leaf, the roots, stems and the midrib and petiole samples of the newest leaf (the top leaf). This finding strongly suggests that CLbr movement *in planta* follows the source to sink relationship as previously described for ‘*Ca.* Liberibacter asiaticus’ (CLas) and ‘*Ca.* Liberibacter solanacearum’ (CLso). No symptoms consistent with Liberibacter-associated diseases were noted for plants colonised by CLbr during this study, consistent with the hypothesis that CLbr does not cause disease of eggplant during the early stages of host colonisation. In addition, no significant differences in biomass were found between eggplant colonised with CLbr, compared to those that were exposed to CLbr-negative *A. solanicola,* and to control plants.

## Introduction

The Liberibacter genus includes both phytopathogenic and non-pathogenic phloem-limited gram-negative alpha-proteobacterial species. Phytopathogenic *Candidatus* Liberibacter species cause devastating diseases globally, in a wide range of host plants belonging to the Rutaceae, Solanaceae and Apiaceae families, affecting both production and trade. The Liberibacter species are predominantly transmitted by psyllids in a circulative persistent manner and can replicate in both their psyllid and plant hosts^1–4^.

A novel candidate Liberibacter species, *‘Candidatus* Liberibacter brunswickensis’ (CLbr) was discovered in *Acizzia solanicola*, commonly known as the eggplant psyllid^5^. CLbr was described as a new Liberibacter species based on multi-locus sequence analyses and is the first species of the Liberibacter genus to be detected in mainland Australia^5^. Host plants of *A. solanicola* (as defined by Burckhardt et al.) include *Solanum melongena* (commonly known as eggplant, brinjal or aubergine), *Solanum mauratianum* (wild tobacco bush), *Physalis peruviana* (cape gooseberry), an undetermined species of *Datura* (thornapple), an undetermined species of *Brugmansia* (angel’s trumpet) and *Solanum pterophilum* (native rock nightshade)^6–8^. No Liberibacter-associated disease symptoms have been reported for the eggplant or wild tobacco bush colonised by *A. solanicola*, and no symptoms were evident upon psyllid collection nor during laboratory colony maintenance (for a range of psyllid infestation levels)^5^.

*‘Candidatus* Liberibacter solanacearum’ (CLso) is the causative agent of zebra chip disease of potato (*Solanum tuberosum)* and yellows or vegetative disorders in various solanaceous and apiaceous crop hosts^4,9–13^. CLso haplotypes A and B are known to be vectored by the tomato potato psyllid, *Bactericera cockerelli,* which shares the host plants with *A. solanicola*, eggplant, wild tobacco bush and cape gooseberry^9,10,14,15^. CLso haplotypes A and B are also able to infect crops including tomatoes (*Solanum lycopersicum*) and capsicum (*Capsicum annuum*)^4,9,10,12^. CLso has been detected in host plants by quantitative polymerase chain reaction (qPCR) within 7–14 days post inoculation and disease symptoms apparent 20–30 days post inoculation^16–18^. Symptoms include yellow to purple foliage discoloration, interveinal chlorosis, spiky appearance of new foliage, proliferation of auxiliary buds with shortened internodes, swollen nodes, aerial tubers (for potatoes) and death of plants^9,12,19^. For eggplant in particular, CLso infection results in leaf chlorosis and cupping, overall stunting and production of small and malformed fruits^20^.

Huanglongbing (HLB) and citrus greening are known as the most destructive diseases of citrus to date and are caused by three phytopathogenic Liberibacter species; ‘*Candidatus* Liberibacter asiaticus’ (CLas), ‘*Candidatus* Liberibacter americanus’ (CLam) and ‘*Candidatus* Liberibacter africanus’ (CLaf)^21–25^. CLas and CLam are predominantly vectored by the Asian citrus psyllid, *Diaphorina citri*, and for CLaf, the African citrus psyllid, *Trioza erytreae. D. citri* and CLas interactions are the most widely studied due to their wide geographic spread and destruction of citrus^23^. Symptoms include yellowing shoots, discoloured blotchy mottled foliage, discoloured and lopsided fruit, premature defoliation and tree stunting leading to early death^21,26^. The appearance of symptoms can appear from 3 months (under the best climate conditions) to years after initial inoculation, which may also vary on the flushing dynamics^24,25,27–30^. Alves et al*.*^31^ assessed CLas titres in new shoots of *Citrus* × *sinensis* (susceptible), *Murraya paniculata* (partially resistant), and *Bergera koenigii* (fully resistant) hosts after inoculation by CLas-positive *D. citri*. CLas was detected as early as 2 days post inoculation access period (DPIAP), followed by a drop in CLas titres until the 10–12 DPIAP for all hosts. The CLas titres increased exponentially before reaching a stationary phase for both *C.* × *sinensis* (to ∼ 5 log Las cells/g of tissue from DPIAP onwards) and *M. paniculata* (to ∼ 3 log Las cells/g of tissue between 40 and 60 DPIAP, then undetectable from the 160 DPIAP)^31^.

The distribution of Liberibacter species within their plant host is via the phloem and can be highly variable depending on various environmental factors^32,33^. Due to their fastidious nature, there are gaps in our understanding of the transmission characteristics (acquisition, latency, infectivity, and inoculation) and pathogenicity mechanisms of the destructive phytopathogenic Liberibacter species^24,34–37^. Various studies on CLso and CLas transmission by *B. cockerelli* and *D. citri,* respectively, have been performed to inform effective management strategies for the psyllid vector populations and to understand plant–insect–microbe interactions^1,17,26,38–40^. Phloem ingestion is required for psyllids to acquire Liberibacter species and evidence of circulative propagative transmission by psyllids has been shown^1,41,42^. Once acquired and before inoculation of the bacterium can occur, a latency period of approximately two weeks is required, during which the bacterial cells replicate within their psyllid host^1,41^. After ingestion, CLso and CLas appear to pass through the midgut epithelium to the haemolymph, here the Liberibacter species can spread to the salivary glands^41–43^. Inoculation of microbes to the phloem can occur during psyllid salivation, once the stylet has reached the phloem tissue^44,45^. Liberibacter inoculation to plant hosts increases when the psyllid has acquired the Liberibacter as a nymph^26,39^.

Few studies have been published on Liberibacter species that are not considered to be the causal agent of agricultural crop diseases, such as *Liberibacter crescens* (Lcr) to papaya, *Ca.* Liberibacter europaeus (CLeu) to pear and *Ca.* Liberibacter ctenarytainae (CLct) (the plant host range based on the psyllid host range, has not been tested)^46–51^. Raddadi et al.^47^ showed the presence of CLeu in high titres in the pear psyllid host, *Cacopsylla pyri* and pear plants. Field collected psyllids positive for CLeu were able to colonise healthy pear plants with no apparent disease expression over 6 months. CLeu has also been detected in both Scotch broom, *Cytisus scoparius,* and the broom psyllid, *Arytainilla spartiophila* in New Zealand^50^. To date, it is unknown if CLeu has a role in controlling scotch broom, or if it can colonise scotch broom (without the presence of the broom psyllid). There is no information published on the temporal or spatial distribution patterns of either Lcr or CLeu during colonisation of host plants and if these patterns are similar to that of the phytopathogenic Liberibacter species. Furthermore, there is no data on the acquisition and inoculation of non-pathogenic Liberibacter species.

This study is the first investigation into the relationships of CLbr with its known psyllid and plant hosts. Therefore, the objective of this work was to determine if CLbr can colonise and persist in eggplant without psyllids feeding, and if so, determine the rate of spread and distribution of CLbr in eggplants, and determine if CLbr colonisation has any effect (positive or negative) on the early development of eggplants. In addition, we investigated the acquisition of CLbr by Liberibacter-negative *A. solanicola* psyllids.

## Results

### CLbr replication and systemic presence in eggplants

**Figure 1:**
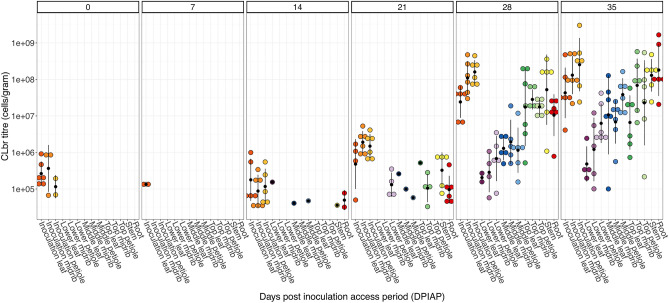
CLbr replication and distribution in early eggplant development. CLbr titres (cells/gram) detected from 14 sample locations in all plants tested were plotted over a 5-week period. Each CLbr-positive sample is plotted (coloured circles) and the mean CLbr titre is indicated (black circles) with standard deviations (black lines). The first timepoint has been labelled 0 days post inoculation access period (DPIAP), as plants were removed from the experiment after the psyllids inoculation access period was complete (3 days in total).

All plants tested prior to the experiment were determined to be CLbr-free (data not shown). CLbr was transmitted to all eggplants by CLbr-positive *A. solanicola* psyllids, with the rate of detection and the number of CLbr cells increasing in the eggplants overtime (Fig. 1, Supplementary Tables 1 and 2). CLbr was not detected in any of the samples taken from the CLbr-negative *A. solanicola* feeding plants, nor by the psyllid-free control plants at any time point.

At the end of the inoculation access period (IAP) (labelled 0 days post (DP) IAP) low levels of CLbr were detected in all leaf samples (100%), half of the midrib samples (50%), and two petiole samples (33%) of the inoculated leaf of plants colonised by the CLbr positive psyllids (Fig. 1). The mean titre of CLbr equated to less than 3 CLbr cells/µL the midrib samples and petiole samples (2.99 × 10^5^ cells/gram and 4.38 × 10^4^ cells/gram respectively) in and approximately 3 CLbr cells/µL (3.45 × 10^5^ cells/gram) in the leaf samples. At 7 DPIAP, CLbr was only detected in leaf samples of the inoculation leaf of two plants (33%) and the mean titre dropped below 5 CLbr cells/µL (4.51 × 10^4^ cells/gram) (Fig. 1 and Supplementary Table 2).

At 14 DPIAP, the number of CLbr positive inoculation leaf samples increased. All inoculation leaf midrib samples were positive for CLbr and 83% of the leaf and petiole samples were CLbr-positive at low levels (Fig. 1 and Supplementary Table 2). Detection of CLbr outside the inoculation leaf occurred at 14 DPIAP in a small number of samples; in the roots (33% of replicates), one stem sample (16%), one petiole and leaf sample of the middle leaf (16%) and one leaf sample of the lower leaf (16%) (Fig. 1 and Supplementary Table 2A). However, the mean titres of the samples that tested positive were at very low levels, equating to less than one CLbr cells/µL (between 5.97 × 10^3^ and 2.57 × 10^4^ cells/g) (Fig. 1 and Supplementary Table 2).

By 21 DPIAP, CLbr was detected in almost all parts of the plant (excluding the petiole samples of the top leaf and the leaf and midrib samples of the lower leaf). All root samples and the majority of stem samples (83%) were positive for CLbr at low levels at 21 DPIAP, with the mean titres approximately one and four CLbr cells/µL (1.40 × 10^5^ and 4.03 × 10^5^ cells/g), respectively. All midrib and petiole samples of the inoculation leaf were positive with the mean CLbr titre increasing tenfold, to approximately 23 and 18 CLbr cells/µL (2.35 × 10^6^ and 1.89 × 10^6^ cells/g) respectively (Fig. 1 and Supplementary Table 2). Approximately 6 CLbr cells/µL (5.69 × 10^5^ cells/g) were detected in the leaf samples (66%) of the inoculation leaf (Fig. 1 and Supplementary Table 2).

All sample locations on eggplants inoculated with CLbr positive psyllids were positive by 28 and 35 DPIAP (Fig. 1 and Supplementary Table 2). High CLbr titres, above 1000 CLbr cells/µL were first reached in the inoculation midrib, inoculation petioles and stems of all plants 28 DPIAP samples (1.71 × 10^8^, 2.00 × 10^8^ and 1.43 × 10^8^ cells/gram, respectively) (Fig. 1 and Supplementary Table 2). CLbr titre increased in samples from the lower and middle leaves over the same timeframe, but not at such a high rate as the stems and roots of the colonised plants. Of the leaf samples tested, CLbr titre was higher in all samples of the top leaf (the newer leaf), when compared to the middle and lower (the older and oldest) leaf samples.

All samples were positive in all plants at the final time point 35 DPIAP, excluding the leaf (63%) and midrib (83%) samples of the lower leaf (Fig. 1 and Supplementary Table 2). The highest mean titres were still seen in the inoculation leaf samples, reaching approximately 7.61 × 10^3^, 2.22 × 10^3^ and 1.14 × 10^3^ CLbr cells/µL (7.61 × 10^8^, 2.22 × 10^8^ and 1.14 × 10^8^ cells/gram) in the petiole, midrib and leaf samples respectively. The mean titre in the root samples increased from 157 CLbr cells/µL (1.57 × 10^7^ cells/gram) at 28 DPIAP to 4.85 × 10^3^ CLbr cells/µL (4.85 × 10^8^ cells/gram) at 35 DPIAP. The mean CLbr titre in the stem and the top leaf samples remained high, increasing to approximately 1.86 × 10^3^ CLbr cells/µL (1.86 × 10^8^ cells/gram) in the stem, 1.69 × 10^3^ CLbr cells/µL (1.69 × 10^8^ cells/gram) in the top leaf midrib and 1.22 × 10^3^ CLbr cells/µL (1.22 × 10^8^ cells/gram) in the top leaf petiole samples. The only sample type that did not increase in the mean number of CLbr cells/µL at 35 DPIAP was the top leaf tissue, which was approximately 800 CLbr cells/µL (8.00 × 10^7^ cells/gram) at 28 DPIAP to 173 CLbr cells/µL (1.73 × 10^7^ cells/gram) at 35 DPIAP (Fig. 1 and Supplementary Table 2).

### Eggplant biomass comparison

**Figure 2:**
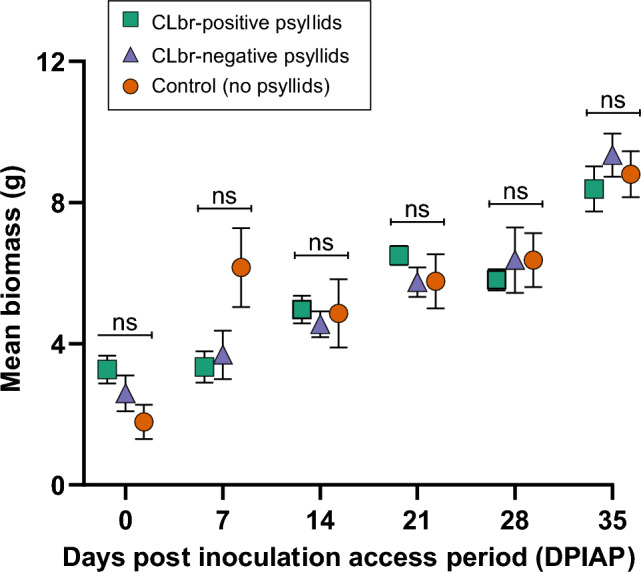
Eggplant biomass (mean ± SEM) at each time point (ns, no significant difference by Fisher LSD test; *p* > 0.05). The mean biomass of all treatments was compared at each timepoint, showing the standard error of the mean.

The mean biomass of eggplants was compared for all treatments at all time points (Fig. 2). Colonisation of eggplants by CLbr or CLbr-negative psyllids did not influence mean plant biomass at any time point (Fig. 2 and Supplementary Table 3). In addition, no significant difference was observed in the change in number of leaves between each treatment or timepoint (Supplementary Table 3). Visually no differences were observed between the treatment groups and by 35 DPIAP, flowers were either present or forming for all treatment groups.

### *A. solanicola* colony acquisition rates of CLbr

The CLbr-negative Bellingen, NSW psyllids were able to acquire CLbr from CLbr-positive eggplants. From the 12th week onwards, all individuals were positive for CLbr (Table 1). The average titre of CLbr varied overtime (Table 1). The highest average CLbr titre, approximately 2.57 × 10^6^ cells/µL, was observed at the first-time point tested, 6 weeks after the colony start (Table 1). However, not all psyllids assessed were positive. The average CLbr titre dropped at 10 weeks after the colony start.

**Table 1:** *Acizzia solanicola* colony acquisition of CLbr.

Weeks after colony start	Number of psyllids (CLbr positive/total)	Percent CLbr positive (%)	Lowest CLbr titre (cells/µL)	Highest CLbr titre (cells/µL)	Average CLbr titre (cells/µL)
6	9/11	82	4.31 × 10^2^	2.31 × 10^7^	2.57 × 10^6^
9	13/22	59	1.52 × 10^2^	4.44 × 10^6^	3.75 × 10^5^
10	9/12	75	2.40 × 10^2^	5.58 × 10^3^	1.92 × 10^3^
12	24/24	100	4.36 × 10^3^	5.43 × 10^5^	8.75 × 10^4^
13	23/23	100	2.82 × 10^4^	5.82 × 10^6^	8.02 × 10^5^
14	23/23	100	4.15 × 10^2^	7.60 × 10^6^	9.92 × 10^5^
20	13/13	100	8.41 × 10^2^	8.57 × 10^6^	1.12 × 10^6^

## Discussion

The novel Liberibacter species, CLbr, was discovered in the psyllid, *A. solanicola* in the absence of plant disease^5^. This study showed that CLbr can be transmitted by *A. solanicola* adult psyllids to eggplant, and further can replicate, move and persist within the eggplant. Likewise, CLbr can be acquired by CLbr-negative *A. solanicola* individuals feeding on CLbr positive eggplants (Supplementary Results). No symptoms consistent with disease were noted on CLbr-positive eggplant plants during early stages of plant development in this study.

After successful transmission from *A. solanicola* to the eggplant via insect feeding, the CLbr titre increased showing that the bacterium was able to replicate *in planta*. A single leaf inoculation site allowed CLbr to be followed as it spread through the plant. CLbr was first detected outside the inoculation leaf at 14 DPIAP at very low titres (Fig. 1, Supplementary Tables 1 and 2). Over time, the titre of CLbr within all tissues of the eggplant increased significantly. The highest titres were detected in all petiole samples of the inoculation leaf and the roots at 7.16 × 10^3^ and 4.83 × 10^3^ CLbr cells/µL (7.16 × 10^8^ and 4.83 × 10^8^ cells/gram) respectively, strongly suggesting that the bacterium is replicating inside the plant. Alves et al.^31^ noted a similar drop in CLas titres in new shoots of susceptible and partially resistant hosts. This was labelled a “dilution effect” due to the growth of new shoots^31^. Like that of CLbr, exponential growth was noted for CLas in both the susceptible and partially resistant hosts, then both reaching a plateau^31^. Longer-term colonisation experiments are required to confirm if CLbr titres have also reached a plateau in eggplants.

Translocation of CLso and CLas have been shown to spread systemically *in planta* with varied titres across tissue types^16,52,53^. The movement of CLso and CLas *in planta* is believed to passively follow sugar transport within the phloem as the bacteria moves from sources (leaves) to sinks (roots, tubers, young leaves, flushes, fruits) in the same way phytosynthates are transported to sinks for storage or use in new growth^16,54–56^. Movement of CLbr throughout eggplant seedlings may also passively follow metabolite and carbohydrate transport in the phloem. Throughout this experiment, eggplant tissue types that are normally regarded as sinks (e.g. roots and young leaves) increased in CLbr titre more rapidly than those that are usually categorised as sources (e.g. mature leaves) in a manner that would support this hypothesis. For example, at 28 DPIAP the newly developed, actively growing leaf tissue collected from the top of the eggplant (leaf, midrib and petiole), roots and stem had higher CLbr titres than the maturing middle and lower leaf samples (Fig. 1 and Supplementary Table 2). Additionally, by 35 DPIAP, CLbr distribution appeared to be systemic as the bacterium was detected in all sample types.

Biomass accumulation in early development of eggplants was not influenced by the colonisation of CLbr. In contrast, CLso infection of potato and tomato plants in the early stage of development typically results in disease symptoms after 3 weeks and some plants died as early as 7-weeks post IAP^16,18,57^. The absence of any detrimental effect on eggplant growth when colonised with CLbr and the production of flowers for most plants by 35 DPIAP, further supports the conclusion that CLbr is not associated with disease on this host. However, longer-term colonisation experiments under varying environmental conditions are required to determine whether CLbr colonisation affects fruit production.

There are limited published data on the replication rates, psyllid acquisition and inoculation of non-pathogenic Liberibacter species. In this study we show that then bacterium can be acquired by CLbr-free *A. solanicola* from a CLbr colonised eggplant. The *A. solanicola* colony CLbr acquisition experiment showed that the number of CLbr-positive psyllids increased overtime and by 12 weeks after the colony start 100% of the *A. solanicola* psyllids that were feeding on the CLbr colonised eggplants were positive for CLbr (Table 1). The CLbr titre and percentage of CLbr-positive *A. solanicola* individuals increased until 10 weeks after the colony start, as at this point new CLbr-negative plants were added to the insect cage to sustain the large colony size. CLbr titres were variable in the *A. solanicola* individuals over the 20-week testing period, which is consistent with published findings on CLso and CLas^38,58^. Higher rates of CLas acquisition and inoculation have been reported when the psyllids have been feeding on infected plants from nymphal stages^34,39^. Further work is needed to determine the feeding patterns and inoculation potential of CLbr by *A. solanicola* individuals and to determine the movement and distribution of CLbr in *A. solanicola*.

## Materials and methods

### Psyllid colonies

Three colonies of *A. solanicola*, one CLbr-positive and two CLbr-negative, were used. The first one was stablished with psyllids originally collected from an eggplant, *S. melongena*, in a residential property in Brunswick, Victoria^5^. The second and third colonies were established with psyllids collected from, respectively, an eggplant in a residential property in Bellingen, New South Wales and a wild tobacco bush, *Solanum mauritianum* plants in Clybucca, New South Wales. Absence/presence of CLbr and other Liberibacter species in the psyllids was made using the Liberibacter generic conventional polymerase chain reaction (PCR)^5^ and a CLbr specific quantitative PCR (qPCR) (described below). Sequence analysis of the mitochondrial genomes of an individual psyllid from each of the three colonies found that *A. solanicola* from NSW share 100% nucleotide identity (Supplementary Methods and Results). The Victorian *A. solanicola* mitochondrial genome shared 100% average nucleotide identity to the NSW colonies, indicating that the colonies were genetically similar (Supplementary Methods and Results).

All colonies were maintained in insect cages (BugDorm, Taiwan) in a controlled environment room on *S. melongena* variety Black Beauty at 20 °C (± 2 °C) and 60% (± 5%) relative humidity, with a photoperiod of 16:8 h (Light:Dark). CLbr status in psyllids from all three colonies was monitored periodically using qPCR (described below).

### Psyllid deoxyribonucleic acid (DNA) extractions

Individual psyllids were homogenised using the Bead-Mill TissueLyser (Qiagen, Germany) and nucleic acids extracted using the Qiagen DNeasy Blood and Tissue Kit (Qiagen, Germany) as described in Morris et al.^5^.

### Plant DNA extractions

Total DNA was extracted from plant material using the Qiagen DNeasy Plant Mini Kit (Qiagen, Germany) according to manufacturer’s instructions with the following amendments; 0.2 g of plant material (for each sample type per plant, described below and presented in Fig. 1) was added to 2 mL of CTAB buffer (2% CTAB, 1.4 M NaCl, 1% PVP-40, 0.02 M EDTA and 0.1 M Tris- HCl, pH 8.0) and homogenised in a universal BioReba extraction bag (BIOREBA AG, Switzerland) using the Homex grinder (BIOREBA AG, Switzerland). A 500 µL aliquot of the homogenate was transferred to a microcentrifuge tube, 4 µL of RNase A added and incubated at 65 °C for 30 min. Qiagen DNeasy Plant Mini Kit instructions were resumed, and two elution steps were performed with a final elution volume of 100 µL. Plant DNA Extraction" using 0.2 g of plant material was performed on eggplants grown in the laboratory from seed, purchased from the garden store.

High throughput total DNA extraction of plant tissues was performed using the MagJET Plant gDNA Flex Kit (Thermo Scientific, Germany) and the KingFisher Flex Purification System (Thermo Scientific, Germany) in 96-well plate format according to the manufacturer’s instructions with the following amendments: Samples were not added to every 15^th^ tube to serve as extraction negative controls for the high-throughput extraction procedure. For mechanical sample lysis a single 3 mm stainless steel bead was added to each 1.4 mL 2D Data-Matrix coded Screw Cap V bottom tube (Micronic, The Netherlands), covered with the rubber plate seal and homogenized using a Bead-Mill TissueLyser (Qiagen, Germany) at 30 megahertz (Mhz) for 1 min. Plates were centrifuged at 13,495 × g for 1 min, rotated, then homogenisation and centrifugation were repeated. Lysis buffers and RNase A was added according to the manufacturer’s instructions, the plate resealed and then mixed by inverting. The extraction resumed according to manufacturer’s instructions with samples eluted in 100 µL.

### Quantitative Polymerase Chain Reaction (qPCR)

To estimate the titre of CLbr in the psyllid and plant extracts, a novel CLbr-specific forward primer, CLbr-F 5’TCGAGCGCGTATGTAAATACG3’, was developed to be used in conjunction with the published HLB-r reverse primer and HLB-p, probe (^59^). The specific primer combination was validated in silico*,* excluding all known species of Liberibacter and other closely related alpha proteobacteria (NCBI numbers and strains described in Morris et al*.*^5^). The primer combination was tested using the AgPath-ID™ One-Step RT-PCR Reagents (ThermoFisher Scientific, United States of America) against DNA extracts positive for CLbr, CLas, CLeu and CLso. The primer combination could exclude the solanaceous Liberibacter species but did amplify CLas DNA extracts. TaqMan™ Ribosomal RNA Control Reagents (ThermoFisher Scientific, United States of America) and the COX primer probe set(^60^) were used as internal controls for the psyllids and plants, respectively. Reactions were performed in 20 µL with 250 nM primer and 150 nM probe concentrations, using 5 µL of template DNA and amplified on the Applied Biosystems Quant Studio 3 System (ThermoFisher Scientific, United States of America) with the following conditions; 10 min at 45 °C, 10 min at 95 °C and 40 cycles of 94 °C for 30 s and 58 °C for 60 s. Ten-fold serial dilutions of cloned 16S rRNA region extracts of CLbr developed in Morris et al.^5^, in UltraPure water (ThermoFisher Scientific, United States of America. The serial dilutions were quantified using the Qubit double stranded DNA (dsDNA) HS assay for the Qubit 2.0 fluorometer (Invitrogen, Germany) and used as a standard curve for estimation of the CLbr titre of 16S rRNA region copy number on the Applied Biosystems Quant Studio 3 System (ThermoFisher Scientific, United States of America). The quantity was calculated per cell, per microliter (cells/µL) of DNA extract and per gram (cells/gram) (Supplementary material and Supplementary Table 1). The means of each sample across all plants at each time points were calculated (Supplementary Table 2). A CLbr negative qPCR result was defined as having a cycle threshold (Cq) value > 35 (Supplementary Material).

### Transmission of CLbr from psyllid to plant

#### Test plants

Nine-week-old *S. melongena,* Black Beauty, were grown in small pots (8.5 cm × 8.5 cm × 12 cm) from seed in the same conditions as the psyllid colonies described above. Plants at the 8–9 leaf stage were selected for the experiment. Plants were maintained in standard potting mix (Bio Gro, Australia) including course vermiculite, course perlite, Macracote Coloniser Plus 4-month slow release fertiliser (15N: 3P: 9 K), nitrogen slow release fertiliser (40N: 0P: 0K), water holding granules, trace elements (6Mg: 6.5Fe: 5.4S: 1.5Mn: 0.4Zn: 0.14B: 0.07Mo) and garden lime. To ensure plants used in the experiments were free from Liberibacter species, a sub sample of plants were tested using generic Liberibacter primers^5^ and CLbr qPCR primers prior to the start of the experiment.

#### CLbr presence, replication in eggplants and eggplant biomass assessment

Sixteen *A. solanicola* individuals from the CLbr-positive colony were aspirated into a 30 mL collection tube, starved for 12 h and transferred to a small (15 cm × 6 cm) insect rearing bag (BugDorm, Taiwan). To act as a negative-CLbr psyllid feeding control, this process was repeated concurrently with a CLbr-negative colony from Bellingen, New South Wales. Psyllids were restricted to an upper leaf, termed “the inoculation leaf”, for a 3-day (72 h) inoculation access period (IAP) and the 16 psyllids were then removed using aspiration and stored at − 80 °C until processing. Plants were sprayed with Confidor (0.125 g/L Imidacloprid) after the inoculation access period, to eliminate the potential presence of any remaining psyllids or viable eggs. Control plants with no psyllids feeding were run concurrently and positioning of all plants in the controlled environment chamber was randomised. Six replicates were performed for each treatment.

Six replicates of each treatment were destructively sampled at six timepoints; the final day of the IAP (once psyllids were removed, labelled day 0), and 7, 14, 21, 28 and 35 days post the inoculation access period (DPIAP). The leaf tissue, midrib and petioles were sampled from three leaves (top, middle and bottom) as well as the inoculation leaf (a single leaf where psyllids were restricted to feeding or an additional leaf for the control plants). In addition, the mid-stem and roots were sampled from each plant. Soil was removed from the roots by shaking, followed by washing in water. Roots were sampled at least 2 cm below the soil surface. Samples of 50 mg (mg) were dissected in duplicate from each plant and stored at − 80 °C until the extraction was performed. Plants were assessed for any Liberibacter-like symptoms on the eggplants throughout the experiment. Leaf numbers were recorded prior to inoculation and prior to destructive sampling as part of the designed experiment. To assess the eggplant biomass, the remaining plant material was dried at 50 °C for a week and the dry mass of each plant at all time points was recorded. The mean biomass in grams (g) was compared for each treatment group at each time point.

#### *A. solanicola* colony acquisition of CLbr

To determine if CLbr-negative *A. solanicola* colonies could acquire CLbr, 50 psyllids from the Bellingen, NSW colony were released into a cage containing two CLbr positive plants. A minimum of 11 psyllids were tested periodically after one-month acquisition access using qPCR. The first sampling point (six weeks post acquisition access) was selected to ensure a new generation of *A. solanicola* were tested. The percentage of CLbr positive psyllids, range of CLbr titre and the average titre at each sample point was determined.

### Statistics

CLbr replication data was analysed and plotted in R version 3.5.1^61^ using ggplot2 version 3.0.0, readr version 1.1.1 and dyplr version 0.7.6^62^. The mean biomass for each time point and treatment (CLbr positive psyllids, CLbr negative psyllids and the Control without psyllids) was calculated from measurements of each eggplant (grams). Each one was submitted to an analysis of variance (ANOVA) in Genstat version 16.1^63^ (Supplementary Table 3). In addition, the mean number of leaves for each time point and treatment calculated form each eggplant at the start of the timepoint, mean number of leaves at the end of that time point and change in leaves (from the start of timepoint to the end of the timepoint) were submitted to an ANOVA^63^ (Supplementary Table 3). The treatment structure was specified by a fully factorial effect for Treatment (CLbr positive psyllids, CLbr negative psyllids and the Control without psyllids) by Time (each time point of the experiment). This was coded in GenStat as Treatment*Time. The blocking structure was specified as the replicates (blocks). This was coded in GenStat as Rep. All residual values were examined graphically to ensure normality and homogeneity of variances. Observations with standardised residuals greater than 3.0 were excluded from analyses. Fishers protected Least Significant Difference (LSD) test (at 0.05 level of significant) were used to separate means when F-tests were significant (Supplementary Table 3). To check the F-probabilities reported in the ANOVA, a permutation test with 4999 iterations was also performed (Supplementary Table 3). In addition, eggplant biomass was plotted using GraphPad Prism version 8.0.0^64^ (Fig. 2). Descriptive statistics was performed for the persistence of CLbr in eggplants and acquisition of CLbr by *A. solanicola*.

### Ethical approval

All collection and experimental research of plant material and insects, comply with relevant institutional and national guidelines and legislation.

### Supplementary Information

Supplementary Information 1.

Supplementary Table 1.

## Data Availability

Data generated and/or analysed during this study are included in this published article or supplementary material. Requests for additional data and code can be sent to the corresponding author. The genomic datasets generated and analysed during the current study are available in the National Centre for Biotechnology Information (NCBI) under the GenBank repository, under the BioProject PRJNA1118906 and accessions listed in supplementary material.
