# Supplementary material for: ‘Candidatus Liberibacter brunswickensis’ colonization has no effect to the early development of Solanum melongena
Source: Sci Rep. 2024 Aug 2;14:17972. doi: 10.1038/s41598-024-66352-y (PMC11297259; doi:10.1038/s41598-024-66352-y)
Supplement: Supplementary file 1 — Supplementary Information 1. [file 41598_2024_66352_MOESM1_ESM.docx]

Supplementary Material

‘*Candidatus* Liberibacter brunswickensis’ colonization has no effect to the early development of *Solanum melongena*

Jacqueline Morris ^1,2,3,4^ *, Rachel Mann ^1,3^, Angage Sanka Perera ^3^, Rebekah Frampton ^1,5^, Mallik Malipatil ^2,3^, Sorn Norng ^3^, Alan Yen ^1,2,3^, Grant Smith ^1,5^, Brendan Rodoni ^1,2,3^ *

^1^Plant Biosecurity Cooperative Research Centre, LPO Box 5012, Bruce, Australian Capital Territory, Australia, 2617
^2^Applied Systems Biology, La Trobe University, AgriBio, 5 Ring Road, Bundoora, Victoria, Australia, 3083
^3^Agriculture Victoria Research, Agriculture Victoria, AgriBio, 5 Ring Road, Bundoora, Victoria, Australia, 3083

^4^Australian Animal Health Laboratories, Australian Centre for Disease Preparedness, Commonwealth Scientific and Industrial Research Organisation (CSIRO), Geelong, Victoria, Australia, 3219

^5^The New Zealand Institute for Plant & Food Research Limited, Gerald St, Lincoln, New Zealand, 7608

*Corresponding Authors: Jacqueline Morris, [jacqui.morris@csiro.au](mailto:jacmmorris@gmail.com) and Brendan Rodoni, [brendan.rodoni@agriculture.vic.gov.au](mailto:brendan.rodoni@agriculture.vic.gov.au)

Supplementary Methods

Quantitative Polymerase Chain Reaction (qPCR) calculations

To calculate the titre of CLbr per cell, the CLbr quantity was divide by three as there is three copies of the 16S rRNA region in Liberibacter genomes. To calculate the number of CLbr cells per microliter (cells/µL) of DNA extract, the CLbr cells value was divided by 5 (as 5 microliters of DNA template was used in the qPCR).

For plant extracts the number of CLbr cells per gram of plant tissues was estimated. The CLbr cells per microliter were multiplied by the initial sample homogenate volume, 2000 µL, to estimate the number of CLbr in 20 mg sample, then multiplied by 50 to estimate CLbr cells per gram (Supplementary Table 1).

Persistence of CLbr in eggplants

To generate CLbr positive plants for psyllid acquisition experiments, opportunistic observations of five eggplants were tested for long-term persistence of CLbr within the plant tissue in the absence of the psyllid host. Approximately 50 CLbr-positive *A. solanicola* individuals were restricted to a single leaf using a small insect net for an inoculation access period (IAP) of 1 week. Plants were sprayed with Confidor one week, and again one month, following the IAP. The midribs and petioles of two leaves were selected for CLbr testing intermittently after 1 month as well as approximately 4 and 6 months after the IAP. The plants were monitored to ensure their psyllid free status was maintained.

Sequence analyses of the mitochondrial genomes

*DNA sequencing*

An individual was selected form each colony from *A. solanicola* from Brunswick, Victoria (VIC1), Bellingen, New South Wales (NSW2) and Clybucca, New South Wales (NSW6). Each DNA extract was quantified using the Qubit dsDNA HS assay and quality estimated using the Nanodrop ND-1000 Spectrophotometer (Thermo Scientific, Germany). Libraries were prepared using the Illumina Nextera XT Library Preparation kit (Illumina Inc, United States of America) following manufacturer’s instructions. The Qubit 2.0 fluorometer and High Sensitivity D1000 protocol on the 2200 TapeStation system (Aligent, United States of America) were used to assess library quality and paired-end sequencing was performed on the HiSeq 2500 as a Rapid Run (2 x 250 base paired reads) (Illumina Inc, United States of America).

*Mitochondrial genome assembly and average nucleotide identity*

Sequence reads were adapter and quality trimmed using TrimGalore (Krueger, 2012) and a *de novo* assembly performed using SPAdes *de novo* version 3.11.0 and the kmer lengths 21, 33, 55 and 77 (Bankevich *et al.*, 2012). A nucleotide BLAST base was made from publicly available psyllid mitochondrial genomes including *Diaphorina citri* (NC_030214.1), *Cacopsylla coccinea* (NC_027087.), *Paratrioza sinica* (NC_02577.1), *Pachpsylla venusta* (NC_006157.1) and *Bactericera cockerelli* (KU051214.1). Nucleotide BLAST version 2.3.0+ (Altschul *et al.*, 1990) was used to identify mitochondrial DNA sequences in each psyllid dataset and a perl script, extractFromFasta.pl was used to extract the mitochondrial genome contigs. Bandage version 0.8.1 was used to assess the *de novo* assembly graph for each psyllid species (Wick *et al.*, 2015). The mitochondrial genome assemblies of *A. solanicola* VIC1 and NSW2 were fragmented. These mitochondrial genomes were closed by mapping to the complete *A. solanicola* NSW6 mitochondrial genome with BWA version  0.7.7 (Li and Durbin, 2009), mapped reads were identified with samtools version 1.2 (Li *et al.*, 2009) and a *de novo* assembly was performed using SPAdes *de novo*. The average nucleotide identity (ANI) was calculated and tabulated using pyani version 0.2.12 (Pritchard, 2016).

Supplementary Results

Persistence of CLbr in eggplant leaves

CLbr was detected in the leaf samples of all eggplants 1 month, 4 months and 6 months post inoculation with an average CLbr titre of 9.21 x 10^4^, 5.91 x 10^5^ and 8.61 x 10^6^ cells/µL respectively (Supplementary Table 4).

Supplementary Tables

Supplementary Table 1. CLbr titres at each sample location across the experiment.

Supplementary Table 2A. Tabulated mean CLbr titre (cells/µL) at each sample location across the experiment.

|  | **Mean CLbr titre (cells/µL) Days Post Inoculation Access Period (DPIAP)** | | | | | |
| --- | --- | --- | --- | --- | --- | --- |
| **Sample Location** | **0** | **7** | **14** | **21** | **28** | **35** |
| Inoculation leaf | 3.45E+00 | 4.51E-01 | 2.93E+00 | 5.69E+00 | 3.33E+02 | 1.14E+03 |
| Inoculation midrib | 2.99E+00 | 0.00 | 1.33E+00 | 2.35E+01 | 1.71E+03 | 2.22E+03 |
| Inoculation petiole | 4.38E-01 | 0.00 | 1.61E+00 | 1.89E+01 | 2.00E+03 | 7.16E+03 |
| Lower leaf | 0.00 | 0.00 | 2.57E-01 | 0.00 | 7.11E-01 | 5.53E+00 |
| Lower midrib | 0.00 | 0.00 | 0.00 | 0.00 | 3.06E+00 | 3.23E+01 |
| Lower petiole | 0.00 | 0.00 | 0.00 | 1.10E+00 | 1.05E+01 | 1.25E+02 |
| Middle leaf | 0.00 | 0.00 | 6.79E-02 | 4.33E-01 | 1.34E+01 | 3.66E+02 |
| Middle midrib | 0.00 | 0.00 | 0.00 | 1.66E-01 | 4.00E+01 | 1.13E+02 |
| Middle petiole | 0.00 | 0.00 | 7.88E-02 | 9.63E-02 | 2.76E+01 | 5.82E+02 |
| Top leaf | 0.00 | 0.00 | 0.00 | 8.65E-01 | 8.00E+02 | 1.73E+02 |
| Top midrib | 0.00 | 0.00 | 0.00 | 9.09E-01 | 3.38E+02 | 1.69E+03 |
| Top petiole | 0.00 | 0.00 | 0.00 | 0.00 | 1.93E+02 | 1.22E+03 |
| Root | 0.00 | 0.00 | 1.82E-01 | 1.40E+00 | 1.57E+02 | 4.85E+03 |
| Stem | 0.00 | 0.00 | 5.97E-02 | 4.03E+00 | 1.43E+03 | 1.86E+03 |

Supplementary Table 2B. Tabulated mean CLbr titre (cells/gram) at each sample location across the experiment.

|  | **Mean CLbr titre (cells/gram) Days Post Inoculation Access Period (DPIAP)** | | | | | |
| --- | --- | --- | --- | --- | --- | --- |
| **Sample Location** | **0** | **7** | **14** | **21** | **28** | **35** |
| Inoculation leaf | 3.45E+05 | 4.51E+04 | 2.93E+05 | 5.69E+05 | 3.33E+07 | 1.14E+08 |
| Inoculation midrib | 2.99E+05 | 0.00 | 1.33E+05 | 2.35E+06 | 1.71E+08 | 2.22E+08 |
| Inoculation petiole | 4.38E+04 | 0.00 | 1.61E+05 | 1.89E+06 | 2.00E+08 | 7.16E+08 |
| Lower leaf | 0.00 | 0.00 | 2.57E+04 | 0.00 | 7.11E+04 | 5.53E+05 |
| Lower midrib | 0.00 | 0.00 | 0.00 | 0.00 | 3.06E+05 | 3.23E+06 |
| Lower petiole | 0.00 | 0.00 | 0.00 | 1.10E+05 | 1.05E+06 | 1.25E+07 |
| Middle leaf | 0.00 | 0.00 | 6.79E+03 | 4.33E+04 | 1.34E+06 | 3.66E+07 |
| Middle midrib | 0.00 | 0.00 | 0.00 | 1.66E+04 | 4.00E+06 | 1.13E+07 |
| Middle petiole | 0.00 | 0.00 | 7.88E+03 | 9.63E+03 | 2.76E+06 | 5.82E+07 |
| Top leaf | 0.00 | 0.00 | 0.00 | 8.65E+04 | 8.00E+07 | 1.73E+07 |
| Top midrib | 0.00 | 0.00 | 0.00 | 9.09E+04 | 3.38E+07 | 1.69E+08 |
| Top petiole | 0.00 | 0.00 | 0.00 | 0.00 | 1.93E+07 | 1.22E+08 |
| Root | 0.00 | 0.00 | 1.82E+04 | 1.40E+05 | 1.57E+07 | 4.85E+08 |
| Stem | 0.00 | 0.00 | 5.97E+03 | 4.03E+05 | 1.43E+08 | 1.86E+08 |

Supplementary Table 3. Summary statistical analyses to compare the mean biomass of eggplants and the mean change in leaf numbers at each timepoint. No significant differences were seen amongst any treatment. F-test Probabilities in brackets are the F-test Probabilities produced from the Permutation Tests.

| **Time** | **Treatment** | **Mean biomass (grams)** | **Mean number of Start Leaves** | **Mean number of End Leaves** | **Change in Leaf number** |
| --- | --- | --- | --- | --- | --- |
| ***0*** | ***Control*** | 1.79 | 8.5 | 8.27 | 0.7 |
|  | ***CLbr+ psy*** | 3.27 | 9.33 | 10.33 | 1 |
|  | ***CLbr- psy*** | 2.59 | 8 | 8.33 | 0.67 |
|  |  |  |  |  |  |
| ***7*** | ***Control*** | 6.16 | 9.33 | 10.4 | 2.47 |
|  | ***CLbr+ psy*** | 3.34 | 8.83 | 12.39 | 3.27 |
|  | ***CLbr- psy*** | 3.69 | 8.83 | 10.96 | 2.72 |
|  |  |  |  |  |  |
| ***14*** | ***Control*** | 4.86 | 9.17 | 9.83 | 3 |
|  | ***CLbr+ psy*** | 4.97 | 10.33 | 16.17 | 5.83 |
|  | ***CLbr- psy*** | 4.56 | 8.17 | 10.17 | 2 |
|  |  |  |  |  |  |
| ***21*** | ***Control*** | 5.77 | 9.67 | 11.67 | 2.33 |
|  | ***CLbr+ psy*** | 6.5 | 9.5 | 12 | 2.83 |
|  | ***CLbr- psy*** | 5.75 | 8.83 | 10.67 | 2.17 |
|  |  |  |  |  |  |
| ***28*** | ***Control*** | 6.37 | 8.17 | 11.33 | 3.17 |
|  | ***CLbr+ psy*** | 5.81 | 9.5 | 11.17 | 2.33 |
|  | ***CLbr- psy*** | 6.37 | 9 | 11.67 | 4 |
|  |  |  |  |  |  |
| ***35*** | ***Control*** | 8.8 | 8.17 | 11.33 | 4.17 |
|  | ***CLbr+ psy*** | 8.39 | 8.83 | 13.17 | 4.33 |
|  | ***CLbr- psy*** | 9.35 | 8.67 | 12.5 | 3.83 |
| **LSD (5%)** | | 1.836 | 1.447 | 3.812 | 3.13 |
|  |  |  |  |  |  |
| **F-Test Probabilities** | |  |  |  |  |
| ***Treatments*** | | 0.759 (0.755) | 0.025 (0.027) | 0.018 (0.057) | 0.487 (0.607) |
| ***Time*** | | <0.001 (<0.001) | 0.353 (0.355) | 0.029 (0.083) | 0.005 (0.021) |
| ***Treatments*Time*** | | 0.128 (0.129) | 0.369 (0.368) | 0.222 (0.459) | 0.465 (0.721) |

Supplementary Table 4A. Persistence of CLbr in eggplants (cells/µL).

| **Months post inoculation** | **Number of eggplants tested** | **Percentage CLbr positive (%)** | **Lowest CLbr titre (cells/µL)** | **Highest CLbr titre (cells/µL)** | **Average CLbr titre (cells/µL)** |
| --- | --- | --- | --- | --- | --- |
| 1 | 5 | 100 | 1.97 E+04 | 2.31 E+07 | 9.21 E+04 |
| 4 | 3 | 100 | 8.64 E+04 | 1.20 E+06 | 5.91 E+05 |
| 6 | 2 | 100 | 7.10 E+06 | 1.01 E+07 | 8.61 E+06 |

Supplementary Table 4B. Persistence of CLbr in eggplants (cells/gram).

| **Months post inoculation** | **Number of eggplants tested** | **Percentage CLbr positive (%)** | **Lowest CLbr titre (cells/gram)** | **Highest CLbr titre (cells/gram)** | **Average CLbr titre (cells/gram)** |
| --- | --- | --- | --- | --- | --- |
| 1 | 5 | 100 | 1.97 E+09 | 2.31 E+12 | 9.21 E+09 |
| 4 | 3 | 100 | 8.64 E+09 | 1.20 E+11 | 5.91 E+10 |
| 6 | 2 | 100 | 7.10 E+11 | 1.01 E+12 | 8.61 E+11 |

**Supplementary Table 5. Distance matrix of the mitochondrial genomes of an *Acizzia solanicona* psyllid from each colony.**

|  | *A. solanicola VIC1 (*PP925602*)* | *A. solanicola NSW2 (*PP925600*)* | *A. solanicola NSW6 (*PP925601*)* |
| --- | --- | --- | --- |
| *A. solanicola VIC1  (*PP925602*)* |  | 100 | 100 |
| *A. solanicola NSW2 (*PP925600*)* | 100 |  | 100 |
| *A. solanicola NSW6 (*PP925601*)* | 100 | 100 |  |

## **References**

Altschul, S. F. *et al.* (1990) ‘Basic local alignment search tool’, *Journal of Molecular Biology*. Academic Press, 215(3), pp. 403–410. doi: 10.1016/S0022-2836(05)80360-2.

Bankevich, A. *et al.* (2012) ‘SPAdes: a new genome assembly algorithm and its applications to single-cell sequencing.’, *Journal of computational biology : a journal of computational molecular cell biology*. Mary Ann Liebert, Inc., 19(5), pp. 455–77. doi: 10.1089/cmb.2012.0021.

Darling, A. E., Mau, B. and Perna, N. T. (2010) ‘progressiveMauve: multiple genome alignment with gene gain, loss and rearrangement.’, *PloS one*, 5(6), p. e11147. doi: 10.1371/journal.pone.0011147.

Guidolin, A. S. and Cônsoli, F. L. (2013) ‘Molecular characterization of Wolbachia strains associated with the invasive Asian citrus psyllid Diaphorina citri in Brazil.’, *Microbial ecology*, 65(2), pp. 475–86. doi: 10.1007/s00248-012-0150-7.

Kent, D. and Taylor, G. (2010) ‘Two new species of Acizzia Crawford (Hemiptera: Psyllidae) from the Solanaceae with a potential new economic pest of eggplant, Solanum melongena’, *Australian Journal of Entomology*, 49(1), pp. 73–81. doi: 10.1111/j.1440-6055.2009.00739.x.

Krueger, F. (2012) *Trim Galore!*, *Babraham Bioinformatics*. Available at: https://github.com/FelixKrueger/TrimGalore/blob/master/Trim_Galore_User_Guide.pdf (Accessed: 26 June 2015).

Li, H. *et al.* (2009) ‘The Sequence Alignment/Map format and SAMtools.’, *Bioinformatics Applications Note*. Oxford University Press, 25(16), pp. 2078–9. doi: 10.1093/bioinformatics/btp352.

Li, H. and Durbin, R. (2009) ‘Fast and accurate short read alignment with Burrows-Wheeler transform’, *Bioinformatics*, 25(14), pp. 1754–1760. doi: 10.1093/bioinformatics/btp324.

Macher, J. N. *et al.* (2018) ‘A simple centrifugation protocol for metagenomic studies increases mitochondrial DNA yield by two orders of magnitude’, *Methods in Ecology and Evolution*, 9(4), pp. 1070–1074. doi: 10.1111/2041-210X.12937.

Martoni, F., Pitman, A. R. and Armstrong, K. F. (2018) ‘DNA Barcoding Highlights Cryptic Diversity in the’, (June). doi: 10.3390/d10030050.

Percy, D. M. *et al.* (2018) ‘Resolving the psyllid tree of life: phylogenomic analyses of the superfamily Psylloidea (Hemiptera)’, *Systematic Entomology*. doi: 10.1111/syen.12302.

Wick, R. R. *et al.* (2015) ‘Bandage: interactive visualization of de novo genome assemblies’, *Bioinformatics*, 31(June), pp. 3350–3352. doi: 10.1093/bioinformatics/btv383.

Wu, F., Cen, Y., Wallis, C. M., *et al.* (2016) ‘The complete mitochondrial genome sequence of Bactericera cockerelli and comparison with three other Psylloidea species.’, *PloS one*. Public Library of Science, 11(5), p. e0155318. doi: 10.1371/journal.pone.0155318.

Wu, F., Cen, Y., Deng, X., *et al.* (2016) ‘The complete mitochondrial genome sequence of Diaphorina citri (Hemiptera: Psyllidae)’, *Mitochondrial DNA Part B*, 1(1), pp. 239–240. doi: 10.1080/23802359.2016.1156491.

Wu, F. *et al.* (2018) ‘Population diversity of Diaphorina citri (Hemiptera: Liviidae) in China based on whole mitochondrial genome sequences.’, *Pest management science*, pp. 1–23. doi: 10.1002/ps.5044.
